# Vitamin D Status During Adolescence and the Impact of Lifestyle Changes: 2 Years’ Follow-up From the Fit Futures Study

**DOI:** 10.1210/clinem/dgad655

**Published:** 2023-11-13

**Authors:** Johanna Öberg, Rolf Jorde, Bjørg Almås, Christopher Sivert Nielsen, Thomas Alexander Gerds, Kevin D Cashman, Guri Grimnes

**Affiliations:** Tromsø Endocrine Research Group, Department of Clinical Medicine, UiT The Arctic University of Norway, N-9037 Tromsø, Norway; Tromsø Endocrine Research Group, Department of Clinical Medicine, UiT The Arctic University of Norway, N-9037 Tromsø, Norway; Haukeland University Hospital, The Hormone Laboratory, N-5021 Bergen, Norway; Department of Chronic Diseases, Norwegian Institute of Public Health, N-0213 Oslo, Norway; Department of Pain Management and Research, Oslo University Hospital, N-0318 Oslo, Norway; Section of Biostatistics, University of Copenhagen, DK-1353 Copenhagen, Denmark; Cork Centre for Vitamin D and Nutrition Research, School of Food and Nutritional Sciences, University College Cork, Cork, Ireland; Tromsø Endocrine Research Group, Department of Clinical Medicine, UiT The Arctic University of Norway, N-9037 Tromsø, Norway; Division of Internal Medicine, University Hospital of North Norway, N-9038 Tromsø, Norway

**Keywords:** vitamin D, adolescence, vitamin d deficiency, longitudinal study

## Abstract

**Context:**

Longitudinal data regarding vitamin D status in adolescence is scarce. This study presents population-based data from an Arctic adolescent population (n = 589) at 16 and 18 years.

**Objective:**

The aims of this study were to investigate changes in vitamin D status during 2 years in adolescence, and whether lifestyle changes were associated with serum 25-hydroxyvitamin D (s-25(OH)D) at follow-up.

**Methods:**

Fit Futures is a longitudinal study at 69°N in Norway. Participants had their s-25(OH)D levels analyzed in their first and third year of upper secondary school (median age 16 and 18 years), in Fit Futures 1 (FF1) and Fit Futures 2 (FF2), respectively. Self-reported lifestyle habits were registered through questionnaires. The association between lifestyle changes and s-25(OH)D levels at follow-up were calculated by regression analyses, controlling for baseline s-25(OH)D levels.

**Results:**

Longitudinal data were available for 309 girls and 280 boys. The proportion of adolescents with s-25(OH)D <50 nmol/L were 73.7% in FF1 and 77.1% in FF2, while the proportion <30 nmol/L constituted 35.7% in FF1 and 40.9% in FF2. Of those with s-25(OH)D <30 nmol/L (severe vitamin D deficiency) in FF1, 73.3% remained severely deficient in FF2. Among boys, an increase in UV exposure was significantly associated with higher s-25(OH)D levels in FF2 (beta; CI [nmol/L] 12.9; 9.1, 16.7). In girls, decreased vitamin/mineral supplement intake was significantly associated with lower s-25(OH)D at FF2 (−6.7; −10.2, −3.1), while increased UV (10.8; 7.0, 14.7) and combined hormonal contraceptive exposure (12.1; 6.0, 18.1) in FF2 was significantly associated with higher s-25(OH)D levels in FF2.

**Conclusion:**

Severe vitamin D deficiency was prevalent throughout adolescence. Lifestyle changes may alter s-25(OH)D levels in this age group.

The measurement of serum 25-hydroxyvitamin D (s-25(OH)D) levels is widely used to evaluate vitamin D status and to guide therapy (1, 2). In observational studies, a single s-25(OH)D measurement is often used to assess the relation between vitamin D status and associated outcomes. However, if vitamin D status changes over time, these results are difficult to apply to long-term outcomes.

Low s-25(OH)D levels in childhood and adolescence are known risk factors for developing rickets and osteomalacia, characterized by the lack of proper mineralization of newly synthesized bone tissue (3). In addition, vitamin D deficiency could lead to impairment of peak bone mass accrual and bone mineral density, although results are inconsistent (4, 5). International guidelines generally agree that s-25(OH)D >50 nmol/L is desirable for good bone health, while levels <30 nmol/L imposes an increased risk of bone-related adverse outcomes (2, 6). There is not full consensus regarding the nonskeletal effects of vitamin D deficiency, but vitamin D is likely involved in numerous disease processes (7).

In North Norway, cutaneous vitamin D production is absent during most winter months (8). In studies of the adult North Norwegian population, correlations between repeated s-25(OH)D measurements are dependent on season and show a clear decline over time (9, 10). A few publications have reported repeated s-25(OH)D measurements in pediatric and adolescent populations (11-13); however, longitudinal data from Norwegian adolescents are missing. Previously published data demonstrated a high prevalence of low vitamin D status in this population (75.8% <50 nmol/L and 38.5% <30 nmol/L), based on single measurements of s-25(OH)D at 16 years (14, 15). If these measurements are predictive of future vitamin D status, early intervention in comparable adolescent populations may be warranted, and identifying population-specific measures to increase s-25(OH)D levels will be of importance.

The aims of this study were to report change in vitamin D status from 16 to 18 years and to investigate the associations between changes in lifestyle factors over the 2 years and s-25(OH)D levels at 18 years.

## Materials and Methods

### Study Population

**Figure 1.**
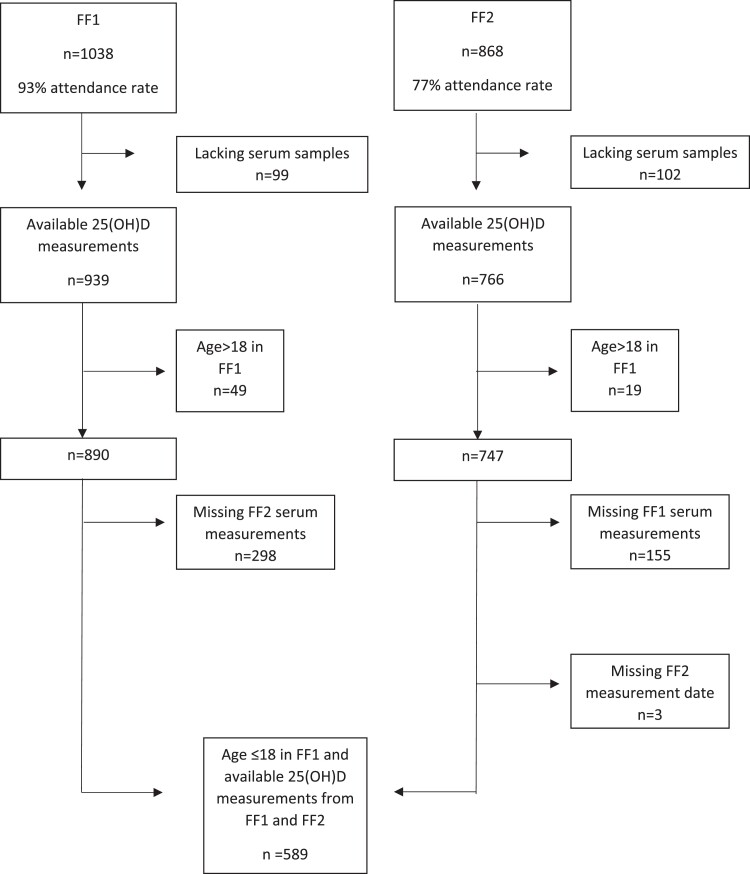
Inclusion process of study participants. Abbreviations: FF1, Fit Futures 1; FF2, Fit Futures 2.

Fit Futures is a population based, longitudinal study in the neighboring municipalities Tromsø and Balsfjord, situated at 69°N. In the first survey, Fit Futures 1 (FF1) from September 2010 to April 2011, all students attending first grade of upper secondary school were invited to participate, while the second survey, Fit Futures 2 (FF2) from November 2012 to June 2013, invited all students in third grade, thus including a majority of those attending FF1. There was no sampling in July and August due to summer holidays. FF1 included 1038 participants (93% attendance), of which 939 persons had available serum samples for analysis. FF2 included 868 participants (77% attendance), with available serum from 766 persons. Participants with age ≤18 years in FF1 and with available serum samples including measurement dates from both surveys were included in this study (n = 589) (Fig. 1). All participants gave written consent before enrolment in the study and participants under the age of 16 years old provided additional consent from their parents/proxy. The Norwegian Data Protection Authority and The Regional Committee for Medical Health Research Ethics North Norway (REK North) approved the study with the project reference 22138/REK North.

### Questionnaire

In this study we included self-reported data reflecting dietary intake of vitamin D (eg, intake of cod liver oil, vitamin/mineral supplements, fatty fish, and extra-semiskimmed milk [fortified with 0.4 µg vitamin D per 100 g]). Further, data reflecting cutaneous vitamin D production by UV exposure (by use of solarium or sunbathing holiday) and puberty status were included. Physical activity, snuff use, and combined hormonal contraceptive (CHC) use among girls were all associated with s-25(OH)D levels in previous publications from this cohort, and thus included in the present study (14, 16).

Pubertal status was categorized by age of menarche in girls (early: <12.5 years, intermediate: 12.5-13.9 years, late: ≥14 years) (17). In boys, the Pubertal Development Scale was used to divide participants in FF1 into the following categories (have not begun, barely started, underway, and completed) (18). Because the Pubertal Development Scale was introduced in the questionnaire after FF1 had started, data for this variable were available in 229 boys, only.

Contraceptive use among girls was assessed by the following questions: “If you have started menstruating, do you use any kind of contraceptives?” (yes/no). “If you use any kind of contraceptives; what types?” (tablets/injections/implants, Implanon/condom/transdermal contraceptive patch/vaginal contraceptive ring/other). Further, users were asked in an interview performed by study nurses about brand name of the contraceptive used and aided by images of the commercial packages for those unable to recall the brand name. This information was used to categorize girls into users of combined hormonal contraceptives (CHC+) and nonusers of combined hormonal contraceptives (CHC–).

Adjustments of some of the variables were needed to secure comparability between FF1 and FF2: In FF1 snuff use was assessed by: “Do you use snuff?” (no, never/sometimes/daily). In FF2, the answer options also included “in the past, but not now;” this answer option was merged with “sometimes.” In FF1 sun-seeking behavior was assessed by the questions: “Have you been on sunbathing holiday/holiday in the south during the last two months?” (yes/no) and “Have you used a solarium during the last 4 weeks?” (yes/no). In FF2 the options were (no/yes, once/yes, several times) here, the last 2 options were recoded as “yes.”

Response categories with few respondents were merged as follows: For the question “How often do you usually drink extra semiskimmed milk?” (Rarely, never/1-6 glasses per week/1 glass per day/2-3 glasses per day/4 or more glasses per day) the last 2 options were recoded into “>1 glass per day.” For the question “How often do you usually eat fatty fish (eg, salmon, trout, mackerel, herring)?” (Rarely, never/1-3 times per month/1-3 times per week/4-6 times per week/every day), the last 2 categories were merged into “4-6 per week or more.”

Lifestyle changes from FF1 to FF2 were categorized into stable (reporting same category in FF1 and FF2), decreased (reporting lower category/less use in FF2) or increased (reporting higher category/more use in FF2). For body mass index (BMI), stable was defined as ±0.5 kg/m^2^ change from baseline (FF1), while a change in BMI larger than +0.5 kg/m^2^ was defined as increased, and a change in BMI larger than −0.5 kg/m^2^ was defined as decreased, from FF1 to FF2. Recent UV exposure was defined as UV exposure in FF2 only and/or UV exposure in both FF1 and FF2.

### Biological Measurements

Nonfasting blood samples were collected at the study site. The samples were stored frozen at −70 °C at the Biobank at UiT—the Arctic University of Norway.

S-25(OH)D was measured by high-pressure liquid chromatography mass spectroscopy at the Hormone Laboratory, Haukeland University Hospital. Measurement of s-25(OH)D_2_ was performed simultaneously with measurement of s-25(OH)D_3_; however, s-25(OH)D_2_ was not detected in the samples, with a limit of detection >10 nmol/L. In 2016, a selection of the samples from FF1 was reanalyzed for s-25(OH)D at the University College Cork, Cork, Ireland, by high-pressure liquid chromatography mass spectroscopy, according to the Vitamin D Standardization Programme protocol for standardization of s-25(OH)D values from past studies. From the standardized s-25(OH)D levels, a statistical regression equation was elaborated and applied to the s-25(OH)D levels (2.6488 + 0.7645 × s-25(OH)D level), thus providing standardized s-25(OH)D levels (15, 19). R^2^ between the original s-25(OH)D levels and the standardized s-25(OH)D levels was 0.98. As the distribution of s-25(OH)D levels in FF2 did not deviate substantially from the levels in FF1, the same regression equation was applied to the s-25(OH)D levels from FF2. The standardized values from both surveys were applied here allowing for a more valid comparison of vitamin D status over the 2 sampling points.

Serum parathyroid hormone (PTH) was measured by electrochemoluminescence immunoassay, using automated clinical chemistry analyzer platforms from Roche Diagnostics. In FF1 measurements were performed consecutively using the Modular E170, and analytical coefficient of variation was 6.4%, 3.7%, and 4.6% at 1.9, 3.4, and 10.1 pmol/L, respectively. The FF2 samples were measured using Cobas 6000, e601. Analytical coefficient of variation was 5% at 5 pmol/L and 3.8% at 3.3 pmol/L. Both surveys measured PTH at the Department of Laboratory Medicine, University Hospital of North Norway, which used Quality Management from Tieto Enator (Helsinki, Finland) for PTH measurements.

### Physical Measurements

Height and weight were measured to the nearest centimeter (cm) and hectogram (hg) without shoes and with light clothing. These values were used to calculate BMI by kg/m^2^.

### Statistics

Baseline characteristics at FF1 were sex stratified and included participants who were ≤18 years in FF1 and had available s-25(OH)D measurements from both FF1 and FF2. Participants were categorized into their vitamin D status according to s-25(OH)D levels, corresponding to international guidelines (<30, 30-50, 50-75, >75 nmol/L) (2, 20).

Stability of vitamin D status was illustrated by spaghetti plots, which for each individual participant connected the s-25(OH)D levels in FF1 and FF2. A multiple linear regression model was used to associate lifestyle changes (cod liver oil intake, vitamin/mineral supplement intake, fatty fish intake, fortified milk intake, physical activity, UV exposure, snuff use, and CHC exposure among girls) with s-25(OH)D levels in FF2 adjusted for baseline s-25(OH)D levels in FF1. Pearson's correlation coefficient was calculated between s-25(OH)D levels in FF1 and FF2.

In additional analysis, we included 1 multiple regression model with change in BMI instead of physical activity (supplementary 1 (21)), and another model which excluded participants with recent UV exposure in FF2 (supplementary 2 (22)). The multiple regression models only included participants with available lifestyle data from FF1 and FF2. The statistical analyses were performed using R (R Core Team (2021) (23).

## Results

**Figure 2.**
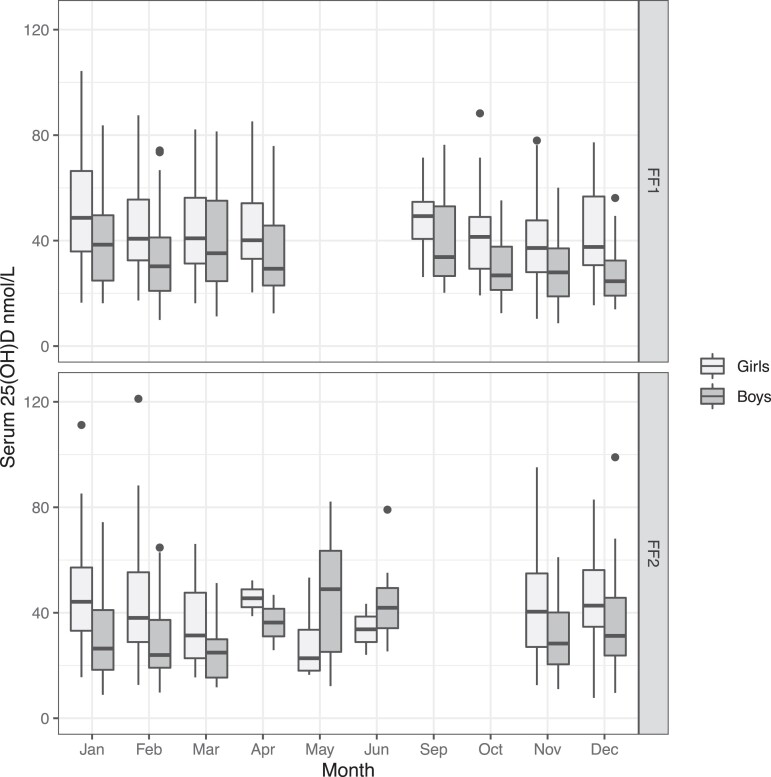
Boxplots with s-25(OH)D nmol/L by attendance month, stratified by sex and survey (FF1 and FF2). Abbreviation: s-25(OH)D, serum 25-hydroxyvitamin D.

Of 589 (97.5% Caucasian) included participants, 309 (52.5%) were girls and 280 were boys (Fig. 1, Table 1, and Table 2). Most serum samples were collected from October to March in both FF1 (86%) and FF2 (95%) and showed no apparent seasonal variation (Fig. 2), therefore no adjustment for season was made.

**Table 1.** Baseline characteristics at FF1 among girls

Variable	Level	s-25(OH)D				Total (n = 309)
		<30 nmol/L (n = 71)	30-50 nmol/L (n = 132)	50-75 nmol/L (n = 86)	>75 nmol/L (n = 20)	
Age (years)	Median (range)	16 (15-18)	16 (15-17)	16 (15-18)	16 (16-17)	16 (15-18)
CHC+	Yes (%)	11 (15.5)	26 (19.7)	26 (30.2)	17 (85.0)	80 (25.9)
s-25(OH)D (nmol/L)	Mean (SD)	24.0 (4.5)	39.7 (5.6)	59.9 (6.4)	84.3 (7.1)	44.6 (17.6)
PTH (pmol/L)	Mean (SD)	4.5 (1.4)	4.0 (1.3)	3.6 (0.9)	3.5 (1.2)	3.9 (1.3)
Puberty status, n (%)	Early	29 (42.0)	46 (35.7)	30 (35.7)	7 (35.0)	112 (37.1)
	Intermediate	25 (36.2)	54 (41.9)	35 (41.7)	10 (50.0)	124 (41.1)
	Late	15 (21.7)	29 (22.5)	19 (22.6)	3 (15.0)	66 (21.9)
BMI (kg/m^2^)	Mean (SD)	22.0 (4.3)	22.0 (3.2)	21.7 (3)	21.2 (2.4)	21.9 (3.4)
Skin type according to sun sensitivity, n (%)	Always red, never brown	5 (7.1)	7 (5.3)	1 (1.2)	0 (0.0)	13 (4.3)
	Almost always red, sometimes brown	17 (24.3)	37 (28.0)	16 (19.3)	3 (15.0)	73 (23.9)
	Almost always brown, sometimes red	32 (45.7)	67 (50.8)	53 (63.9)	15 (75.0)	167 (54.8)
	Always brown, never red	16 (22.9)	21 (15.9)	13 (15.7)	2 (10.0)	52 (17.0)

Data to compute puberty status were available in 302 participants, while skin type was reported by 305 girls.

Abbreviations: BMI, body mass index; PTH, parathyroid hormone; s-25(OH)D, serum 25-hydroxyvitamin D.

**Table 2.** Baseline characteristics at FF1 among boys

Variable	Level	s-25(OH)D				Total (n = 280)
		<30 nmol/L (n = 139)	30-50 nmol/L (n = 92)	50-75 nmol/L (n = 42)	>75 nmol/L (n = 7)	
Age (years)	Median (range)	16 (15-18)	16 (15-17)	16 (15-17)	17 (16-17)	16 (15-18)
s-25(OH)D (nmol/L)	Mean (SD)	22.1 (5)	39.5 (5.8)	58.8 (7.4)	78.5 (3.1)	34.7 (15.9)
PTH (pmol/L)	Mean (SD)	4.5 (1.6)	4.3 (1.5)	4.0 (1.7)	4.2 (0.7)	4.4 (1.6)
Puberty status, n (%)	Has not started	0 (0.0)	0 (0.0)	0 (0.0)	0 (0.0)	0 (0.0)
	Barely started	20 (18.0)	13 (16.9)	7 (20.0)	0 (0.0)	40 (17.5)
	Underway	80 (72.1)	55 (71.4)	26 (74.3)	6 (100.0)	167 (72.9)
	Completed	11 (9.9)	9 (11.7)	2 (5.7)	0 (0.0)	22 (9.6)
BMI (kg/m^2^)	Mean (SD)	22.5 (4.5)	22.2 (3.8)	21.6 (3.1)	21.0 (1.8)	22.2 (4)
Skin type according to sun sensitivity, n (%)	Always red, never brown	11 (8.3)	1 (1.1)	1 (2.4)	0 (0.0)	13 (4.8)
	Almost always red, sometimes brown	30 (22.7)	20 (22.5)	6 (14.6)	1 (14.3)	57 (21.2)
	Almost always brown, sometimes red	67 (50.8)	54 (60.7)	27 (65.9)	3 (42.9)	151 (56.1)
	Always brown, never red	24 (18.2)	14 (15.7)	7 (17.1)	3 (42.9)	48 (17.8)

Data to calculate puberty status were available in 229 participants, while skin type was reported by 267 boys.

Abbreviations: BMI, body mass index; PTH, parathyroid hormone; s-25(OH)D, serum 25-hydroxyvitamin D.

At baseline (FF1), median age was 16 (range 15-18) years and mean BMI was within the normal weight range in both sexes (Tables 1 and 2). Of the girls, 25.9% were CHC + in FF1 (Table 1), in contrast to 46.6% in FF2 (n = 144). Lifestyle variables at baseline (FF1) according to vitamin D status and stratified by sex are presented in Tables 3 and 4.

**Table 3.** Baseline characteristics of modifiable lifestyle variables at FF1 among girls

Variable	Level	s-25(OH)D				Total
		<30 nmol/L	30-50 nmol/L	50-75 nmol/L	>75 nmol/L	
N		71	132	86	20	309
Cod liver oil intake, n (%)	Yes, daily	41 (58.6)	55 (42.0)	32 (37.2)	9 (45.0)	137 (44.6)
	Sometimes	25 (35.7)	53 (40.5)	27 (31.4)	8 (40.0)	113 (36.8)
	No	4 (5.7)	23 (17.6)	27 (31.4)	3 (15.0)	57 (18.6)
	Missing	1	1	0	0	2
Vitamin/mineral supplements intake, n (%)	Yes, daily	34 (48.6)	43 (33.1)	17 (19.8)	6 (30.0)	100 (32.7)
	Sometimes	30 (42.9)	65 (50.0)	35 (40.7)	9 (45.0)	139 (45.4)
	No	6 (8.6)	22 (16.9)	34 (39.5)	5 (25.0)	67 (21.9)
	Missing	1	2	0	0	3
Fatty fish intake, n (%)	Rarely/never	12 (16.9)	20 (15.4)	15 (17.6)	3 (15.0)	50 (16.3)
	1-3 times per month	36 (50.7)	61 (46.9)	44 (51.8)	15 (75.0)	156 (51.0)
	Weekly fish	23 (32.4)	49 (37.7)	26 (30.6)	2 (10.0)	100 (32.7)
	Missing	0	2	1	0	3
Extra-semiskimmed milk intake, n (%)	Rarely/never	39 (56.5)	62 (48.1)	35 (40.7)	6 (30.0)	142 (46.7)
	1-6 glasses per week	12 (17.4)	30 (23.3)	26 (30.2)	3 (15.0)	71 (23.4)
	1 glass per day	12 (17.4)	24 (18.6)	11 (12.8)	6 (30.0)	53 (17.4)
	>1 glass per day	6 (8.7)	13 (10.1)	14 (16.3)	5 (25.0)	38 (12.5)
	Missing	2	3	0	0	5
Physical activity, n (%)	Sedentary	19 (26.8)	11 (8.4)	6 (7.0)	1 (5.0)	37 (12.0)
	>4 hours a week	39 (54.9)	52 (39.7)	25 (29.1)	7 (35.0)	123 (39.9)
	Recreational sports	10 (14.1)	46 (35.1)	28 (32.6)	7 (35.0)	91 (29.5)
	Hard training	3 (4.2)	22 (16.8)	27 (31.4)	5 (25.0)	57 (18.5)
	Missing	0	1	0	0	1
Sunbathing holiday last 2 months, n (%)	Yes	2 (2.8)	8 (6.1)	13 (15.7)	2 (10.0)	25 (8.2)
	No	69 (97.2)	124 (93.9)	70 (84.3)	18 (90.0)	281 (91.8)
	Missing	0	0	3	0	3
Solarium use last 4 weeks, n (%)	Yes	1 (1.4)	33 (25.0)	41 (49.4)	17 (85.0)	92 (30.1)
	No	70 (98.6)	99 (75.0)	42 (50.6)	3 (15.0)	214 (69.9)
	Missing	0	0	3	0	3
Snuff use, n (%)	No, never	52 (73.2)	104 (79.4)	59 (68.6)	4 (20.0)	219 (71.1)
	Sometimes	7 (9.9)	11 (8.4)	16 (18.6)	10 (50.0)	44 (14.3)
	Daily	12(16.9)	16(12.2)	11 (12.8)	6 (30.0)	45 (14.6)
	Missing	0	1	0	0	1

Abbreviation: s-25(OH)D, serum 25-hydroxyvitamin D.

**Table 4.** Baseline characteristics of modifiable lifestyle variables at FF1 among boys

Variable	Level	s-25(OH)D				Total
		<30 nmol/L	30-50 nmol/L	50-75 nmol/L	>75 nmol/L	
N		139	92	42	7	280
Cod liver oil intake, n (%)	Yes, daily	83 (60.1)	40 (44.0)	7 (17.9)	1 (14.3)	131 (47.6)
	Sometimes	43 (31.2)	31 (34.1)	17 (43.6)	3 (42.9)	94 (34.2)
	No	12 (8.7)	20 (22.0)	15 (38.5)	3 (42.9)	50 (18.2)
	Missing	1	1	3	0	5
Vitamin/mineral supplements intake, n (%)	Yes, daily	76 (54.7)	27 (30.0)	11 (28.2)	0 (0.0)	114 (41.5)
	Sometimes	53 (38.1)	37 (41.1)	13 (33.3)	4 (57.1)	107 (38.9)
	No	10 (7.2)	26 (28.9)	15 (38.5)	3 (42.9)	54 (19.6)
	Missing	0	2	3	0	5
Fatty fish intake, n (%)	Rarely/never	38 (27.5)	16 (17.6)	6 (15.0)	0 (0.0)	60 (21.7)
	1-3 times per month	63 (45.7)	36 (39.6)	18 (45.0)	3 (42.9)	120 (43.5)
	Weekly fish	37 (26.8)	39 (42.9)	16 (40.0)	4 (57.1)	96 (34.8)
	Missing	1	1	2	0	4
Extra-semi skimmed milk intake, n (%)	Rarely/never	81 (58.3)	33 (36.7)	22 (55.0)	3 (42.9)	139 (50.4)
	1-6 glasses per week	26 (18.7)	23 (25.6)	5 (12.5)	0 (0.0)	54 (19.6)
	1 glass per day	17 (12.2)	14 (15.6)	5 (12.5)	2 (28.6)	38 (13.8)
	>1 glass per day	15 (10.8)	20 (22.2)	8 (20.0)	2 (28.6)	45 (16.3)
	Missing	0	2	2	0	4
Physical activity, n (%)	Sedentary	52 (37.4)	18 (19.8)	3 (7.5)	0 (0.0)	73 (26.4)
	>4 hours a week	35 (25.2)	23 (25.3)	8 (20.0)	0 (0.0)	66 (23.8)
	Recreational sports	35 (25.2)	20 (22.0)	14 (35.0)	3 (42.9)	72 (26.0)
	Hard training	17 (12.2)	30 (33.0)	15 (37.5)	4 (57.1)	66 (23.8)
	Missing	0	1	2	0	3
Sunbathing holiday last 2 months, n (%)	Yes	5 (3.7)	6 (6.7)	7 (17.1)	3 (42.9)	21 (7.7)
	No	130 (96.3)	83 (93.3)	34 (82.9)	4 (57.1)	251 (92.3)
	Missing	4	3	1	0	8
Solarium use last 4 weeks, n (%)	Yes	5 (3.7)	19 (21.6)	11 (26.8)	3 (42.9)	38 (14.0)
	No	130 (96.3)	69 (78.4)	30 (73.2)	4 (57.1)	233 (86.0)
	Missing	4	4	1	0	9
Snuff use, n (%)	No, never	83 (60.1)	59 (64.8)	33 (82.5)	4 (57.1)	179 (64.9)
	Sometimes	15 (10.9)	13 (14.3)	3 (7.5)	0 (0.0)	31 (11.2)
	Daily	40 (29.0)	19 (20.9)	4 (10.0)	3 (42.9)	66 (23.9)
	Missing	1	1	2	0	4

Abbreviation: s-25(OH)D, serum 25-hydroxyvitamin D.

In FF2, median age was 18 (range 17-21) years. In the total sample, mean s-25(OH)D levels decreased from 39.9 ± 17.5 nmol/L in FF1 to 37.3 ± 17.6 nmol/L in FF2 (Table 5). In FF2, 77.1% had s-25(OH)D levels <50 nmol/L (vitamin D insufficiency) and 40.9% had s-25(OH)D levels <30 nmol/L (severe vitamin D deficiency), compared with 73.7% and 35.7% in FF1, respectively. Stratified by sex, the same trend was seen with declining mean s-25(OH)D levels from FF1 to FF2. Girls with vitamin D insufficiency constituted 65.7% in FF1 compared with 68.6% in FF2, this was true for 82.5% of boys in FF1 compared with 86.4% in FF2. Further, 23.0% of girls had severe vitamin D deficiency in FF1, compared with 26.9% in FF2. In boys, 49.6% had severe vitamin D deficiency in FF1 compared with 56.4% in FF2 (Table 5). The correlation coefficients between s-25(OH)D levels in FF1 and FF2 in the total sample were r = 0.59 (CI 0.54, 0.64), while in girls r = 0.55 (CI 0.47, 0.62) and in boys r = 0.54 (CI 0.45, 0.62) (all *P* < .001). There was no difference when including only those with same sampling month in both FF1 and FF2 (n = 198), r = 0.58 (CI 0.48, 0.68), *P* < .001.

**Table 5.** Mean s-25(OH)D levels and categories of vitamin D status in FF1 and FF2, stratified by sex

Mean s-25(OH)D nmol/L ± SD		FF1			FF2		
		Girls	Boys	Total	Girls	Boys	Total
		44.6 ± 17.6	34.7 ± 15.9	39.9 ± 17.5	42.8 ± 17.7	31.3 ± 15.4	37.3 ± 17.6
s-25(OH)D	<30 nmol/L, n (%)	71 (23.0)	139 (49.6)	210 (35.7)	83 (26.9)	158 (56.4)	241 (40.9)
	30-50 nmol/L, n (%)	132 (42.7)	92 (32.9)	224 (38.0)	129 (41.7)	84 (30.0)	213 (36.2)
	50-75 nmol/L, n (%)	86 (27.8)	42 (15.0)	128 (21.7)	86 (27.8)	35 (12.5)	121 (20.5)
	>75 nmol/L, n (%)	20 (6.5)	7 (2.5)	27 (4.6)	11 (3.6)	3 (1.1)	14 (2.4)

Girls, n = 309; boys, n = 280.

Abbreviation: s-25(OH)D, serum 25-hydroxyvitamin D.

**Figure 3.**
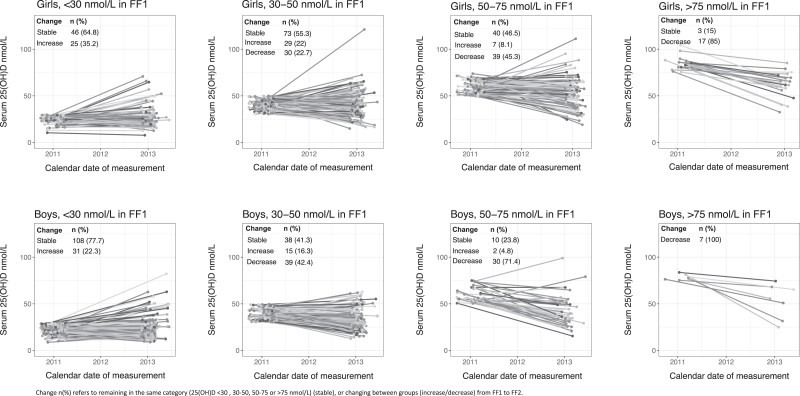
S-25(OH)D levels and change in vitamin D status from FF1 to FF2, stratified by sex and initial vitamin D status in FF1. Abbreviation: s-25(OH)D, serum 25-hydroxyvitamin D.

The spaghetti plots (Fig. 3) presents s-25(OH)D levels from FF1 to FF2, according to vitamin D status in FF1 and sex, to allow for visual interpretation of the data (24). In general, the trends were not sex specific. Most participants with severe vitamin D deficiency did not improve their vitamin D status in FF2, so that 73.3% remained in the severely deficient group, while 49.6% remained insufficient from FF1 to FF2. (Figure 3).

**Figure 4.**
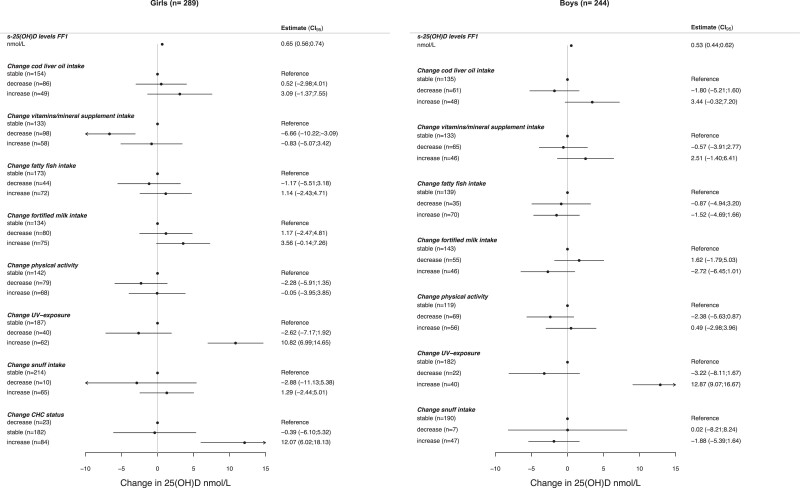
Sex stratified multiple regression model of predicted s-25(OH)D levels in FF2, controlled for baseline s-25(OH)D (FF1) and adjusted for lifestyle variables. Abbreviations: CHC, combined hormonal contraceptives; s-25(OH)D, serum 25-hydroxyvitamin D.

Change in lifestyle factors over the 2 years associated with altered s-25(OH)D at FF2 are presented in a forest plot (Fig. 4). Among girls, a decrease in vitamin/supplement intake from FF1 to FF2 was significantly associated with lower s-25(OH)D levels in FF2 (beta; CI [nmol/L] −6.7; −10.3, −3.3), while increased UV exposure (10.8; 7.0, 14.7) and CHC exposure (starting CHC between FF1 and FF2) (12.1; 6.0, 18.1) were associated with higher s-25(OH)D levels in FF2 (n = 289). Among boys, reporting increased UV exposure in FF2 was associated with higher s-25(OH)D levels in FF2 (12.9; 9.1, 16.7) (n = 244) (Fig. 4).

We chose to only include physical activity in the main analysis, as physical activity and not BMI was associated with s-25(OH)D levels in this cohort previously (14). In the BMI model, among boys, increased intake of cod liver oil was borderline significantly associated with higher s-25(OH)D levels in FF2 (3.87; 0.03, 7.72). The other estimates were not significantly altered (<1 nmol/L) (21).

Assuming that recent UV exposure through solarium use or sunbathing holidays would not necessarily reflect habitual sun exposure, we excluded participants with recent UV exposure in FF2 (22). In this model, among boys, an increase of cod liver oil intake was associated with 5.0 nmol/L higher s-25(OH)D levels (CI 1.0, 9.2, *P* = .02) in FF2, and an increased vitamin/supplement intake was associated with 5.7 nmol/L higher levels (CI 1.2, 10.2, *P* = .01) (n = 187). Among girls (n = 169), no new lifestyle factors were associated with s-25(OH)D levels, but the existing estimates changed. In this model, a decrease in vitamin/mineral intake was associated with −8.4 nmol/L lower s-25(OH)D levels (CI −12.7, −4.2, *P* < .01), while increased CHC exposure (starting CHC between FF1 and FF2) was associated with 16.2 nmol/L higher s-25(OH)D levels (CI 8.4, 24.0, *P* < .01) (22).

Those who did not reattend in FF2 included 298 persons (103 girls and 195 boys) with mean s-25(OH)D levels of in FF1 42.3 ± 18.2 nmol/L and 32.0 ± 15.2 nmol/L for girls and boys, respectively.

## Discussion

The majority of North Norwegian adolescents were severely vitamin D deficient or insufficient both at 16 and 18 years and most severely deficient participants at baseline had not improved their vitamin D status 2 years later. On the contrary, mean s-25(OH)D levels in the cohort decreased during the 2 years, with a larger proportion having levels <50 nmol/L in FF2. Increased UV exposure in both sexes, in addition to increased CHC exposure (starting CHC between FF1 and FF2) among girls, were significantly associated with higher s-25(OH)D levels at FF2, while a decrease in vitamin/supplement intake was associated with lower s-25(OH)D levels among girls. These results indicate that a large part of this adolescent population (only excluding those with recent UV exposure) has lower than recommended vitamin D status.

We have previously reported a high prevalence of vitamin D deficiency during the school year in this population at 16 years (14). After the conclusion of FF1, all participants with s-25(OH)D <25 nmol/L, the limit of vitamin D deficiency in Norway at the time (25) in FF1 received a written information letter with their results and recommendation of supplementation. We expected this to reduce the prevalence of vitamin D deficiency in FF2; however, the opposite was true. The deficiency prevalence is substantially higher than previously reported in other Norwegian adolescent populations, including a recent study of children and adolescents in the south of Norway, where only 47.5% of adolescents had s-25(OH)D levels <50 nmol/L (n = 59), with 66% of the total samples drawn in winter months (26). The vitamin D status in our study is also poorer than in other comparable adolescent populations in Nordic and European countries (27-29).

Despite the possible impact of the sampling design not including summer months, there was a clear difference in prevalence of low vitamin D status between adolescents and adults in North Norway. The prevalence of standardized s-25(OH)D <30 nmol/L in FF1 (median age 16 years) was previously compared with that for adults (mean age 58 years) from Tromsø, North Norway (39.6% vs 0.9% respectively), as part of an EU-funded project (15). The present work highlights how the prevalence of severe vitamin D deficiency (40.9%) in FF2 is also excessively high compared with that of adults in the same location.

In the adult North Norwegian population, there has generally been reported high rates of vitamin D sufficiency (>50 nmol/L), even in studies not including summer months (30, 31). This is likely related to a vitamin D–promoting lifestyle among adults, including more frequent sunbathing holidays and, importantly, sufficient dietary intake of vitamin D (30, 31). The latter is illustrated by a low prevalence of vitamin D deficiency even in the absence of sunbathing holiday sand solarium use among adults (31). Higher adult intake of the traditional “Mølje”-meal, consisting of cod liver and roe, which has a very high vitamin D content, and supplements and fatty fish intake are plausible explanations for the observed age difference (31). This suggests lifestyle differences as the primary cause of the discrepancies in vitamin D status.

Prevention strategies in Norway are aimed at improving vitamin D status in the deficient part of the population. This is implemented by national food fortification strategies and a recommended daily intake of 10 µg of vitamin D (by supplement or cod liver oil), for those with a low vitamin D_3_ diet and little sunshine exposure, and 20 µg in elderly people (>75 years) (32). According to the Nordic Nutrition Recommendations, the daily recommended intake of 10 µg presupposes some vitamin D contribution from natural sunlight exposure. Among those with little or no sun exposure, 20 µg of vitamin D is the recommended intake (33). In our study, over half of the vitamin D–deficient participants reported daily intake of cod liver oil and vitamin/mineral supplements and little recent UV exposure. These results could support that a higher intake is needed in a population with little sun exposure.

As expected, increased UV exposure from FF1 to FF2 was significantly associated with increased s-25(OH)D levels in FF2. Advising tanning as a measure to prevent vitamin D deficiency is not recommended due to increased risk of skin cancer, especially in young people (34). Although moderate UV radiation is known to increase or maintain s-25(OH)D levels (35), dietary sources of vitamin D are the keys to maintain s-25(OH)D levels in those with low sun exposure, including residents in North Norway and polar regions (31, 36, 37). For instance, data from a specifically designed study to estimate the vitamin D dietary requirement in 14-18 year olds at >50°N suggest that a vitamin D intake of 13 μg/day is needed to maintain s-25(OH)D levels ≥30 nmol/L during winter (38). In our previous publication, we found that UV exposure modified the association between s-25(OH)D levels and dietary sources of vitamin D (14). When excluding recent UV exposure in FF2, changes in supplement intake were significantly associated with alterations in s-25(OH)D levels in both sexes, and increased cod liver oil intake was significantly associated with increased s-25(OH)D levels among boys. This emphasizes the significance of dietary sources in persons with low cutaneous vitamin D production.

Severe vitamin D deficiency was more prevalent in boys than in girls at both 16 and 18 years (14). In a previous study using FF1 data, CHC+ was associated with approximately 10 nmol/L higher s-25(OH)D levels among girls, which could explain some of the sex differences (16). The higher s-25(OH)D levels in CHC+ is likely caused by the estrogenic compound in CHC stimulating hepatic vitamin D binding protein production and thus raising s-25(OH)D levels (39, 40). Interestingly, the decline in mean s-25(OH)D levels among girls from FF1 to FF2 was observed despite an increase in CHC+ from 25.2% in FF1 to 45.3% in FF2. However, we did not take into consideration estrogen doses and also lacked data regarding the timing of CHC initiation (41, 42). There was no change in s-25(OH)D levels in past or persistent users of CHC, in accordance with previous studies (43, 44).

This study has some limitations. Firstly, information regarding loss to follow-up in FF2 was not available, which could range from sick leave or unwillingness to participate to apprenticeship for vocational subjects. The lower vitamin D status at baseline in the group not reattending FF2, suggests that the prevalence of severe vitamin D deficiency could be even higher at 18 years in the general population than reported here. On the other hand, as this study largely did not include summer months, the s-25(OH)D levels in the summer season is likely higher than reported in this study. The generalizability of our results to adolescent populations further south has limitations due to latitude differences.

Second, the questionnaire did not contain sufficient dietary information to assess total daily vitamin D_3_ intake. This would have enabled comparison with s-25(OH)D levels to assess the efficacy of the national vitamin D recommendations. Further, we did not have available data on calcium intake. The close interplay between calcium and vitamin D is central to vitamin D metabolism as low calcium intake negatively affect vitamin D balance by increasing vitamin D turnover and inactivation (45). However, the most recent national dietary survey in Norway from 2015 among 13 year olds, found an average ± SD daily calcium intake of 918 mg ± 433 in boys and 753 mg ± 332 in girls (46), close to current recommendations (33).

Third, association with clinical endpoints to evaluate the possible consequences of vitamin D deficiency in this population were not a part of this study and limits the clinical utility of the study results. Studies specifically designed for this purpose, addressing both short-term and long-term consequences are under planning and will address this important question.

This study also has several strengths. It includes longitudinal data from a large, population representative adolescent sample. This is the first study to report longitudinal data on s-25(OH)D levels in the Norwegian adolescent population, thus providing important knowledge of the long-term vitamin D status in this part of the population. Further, we had the possibility to include information regarding change in vitamin D–related lifestyle variables associated with altered s-25(OH)D levels. Another strength is the standardization of s-25(OH)D measurements by the Vitamin D Standardization Programme protocol.

### Conclusion

Severe vitamin D deficiency and insufficiency were highly prevalent in this North Norwegian adolescent population, in fact with a slight deterioration from 16 to 18 years. This calls for improved strategies for avoiding severe vitamin D deficiency in this population.

## Data Availability

Restrictions apply to the availability of some or all data generated or analyzed during this study to preserve patient confidentiality or because they were used under license. The corresponding author will on request detail the restrictions and any conditions under which access to some data may be provided.

## Abbreviations

BMI: body mass index
CHC: combined hormonal contraceptive
FF1: Fit Futures 1
FF2: Fit Futures 2
PTH: parathyroid hormone
s-25(OH)D: serum 25-hydroxyvitamin D
